# Accelerating small-angle scattering experiments on anisotropic samples using kernel density estimation

**DOI:** 10.1038/s41598-018-37345-5

**Published:** 2019-02-06

**Authors:** Kotaro Saito, Masao Yano, Hideitsu Hino, Tetsuya Shoji, Akinori Asahara, Hidekazu Morita, Chiharu Mitsumata, Joachim Kohlbrecher, Kanta Ono

**Affiliations:** 1 Laboratory for Neutron Scattering and Imaging, Paul Scherrer Institute, Villigen, 5232 Switzerland; 2 Toyota Motor Corporation, Toyota, 471-8572 Japan; 3 The Institute of Statistical Mathematics, Tachikawa, 190-8562 Japan; 4 Hitachi Ltd., Tokyo, 100-8280 Japan; 5 National Institute for Materials Science, Tsukuba, 305-0047 Japan; 6 Institute of Materials Structure Science, High Energy Accelerator Research Organization, Tsukuba, 305-0801 Japan

## Abstract

We propose a method to accelerate small-angle scattering experiments by exploiting spatial correlation in two-dimensional data. We applied kernel density estimation to the average of a hundred short scans and evaluated noise reduction effects of kernel density estimation (smoothing). Although there is no advantage of using smoothing for isotropic data due to the powerful noise reduction effect of radial averaging, smoothing with a statistically and physically appropriate kernel can shorten measurement time by less than half to obtain sector averages with comparable statistical quality to that of sector averages without smoothing. This benefit will encourage researchers not to use full radial average on anisotropic data sacrificing anisotropy for statistical quality. We also confirmed that statistically reasonable estimation of measurement time is feasible on site by evaluating how intensity variances improve with accumulating counts. The noise reduction effect of smoothing will bring benefits to a wide range of applications from efficient use of beamtime at laboratories and large experimental facilities to stroboscopic measurements suffering low statistical quality.

## Introduction

Recent progress in materials science using computational approaches such as machine learning and related methods is remarkable^1^. Notably, research involving numerical calculations benefits from those methods because of its intrinsic compatibility with computers. For example, screening a database of numerical calculation results, bypassing heavy calculation, and predicting functional materials from fundamental physical properties of elements^2–5^. However, data-driven materials science which is expected to accelerate material discovery cycles cannot be established only by numerical calculations, but also needs a sufficient amount of experimental results. Filling blank entries in a database with experimental results often requires substantial efforts in sample synthesis, measurement, and analysis. Making these processes more efficient is a key to data-driven materials science. Among various fundamental physical properties and characteristics necessary for data-driven materials science, crystal structures (or phases) and microstructures are essential descriptors for many cases. A combination of combinatorial sample synthesis and high-throughput phase determination using synchrotron x-ray diffraction is one of the promising solutions for the demand^6,7^. On the other hand, compared to rather fast development in high-throughput phase determination for both experimental and data analysis processes, such efforts have been made only for analytical processes for the case of microstructure research^8–11^ because it heavily depends on microscopic techniques which are inherently incompatible with high-throughput measurement. Since microstructure is a crucial factor connecting a broad set of synthesis parameters with beneficial physical properties, efficient microstructural research is as worth as high-throughput phase determination.

Microscopic techniques are not the only methods for microstructure analysis. Small-angle scattering (SAS) contains the bulk average of microstructural information and can complement microscopic surface observation. SAS experiments are divided into two categories: those performed in laboratories using x-ray sources (lab small-angle x-ray scattering, or lab SAXS) and those performed at large experimental facilities using synchrotron x-ray (synchrotron SAXS) or neutron (small-angle neutron scattering, or SANS). Lab SAXS is available at many universities and institutes, but measurement time is usually long due to weak beam intensity of x-ray sources. Synchrotron SAXS and SANS are only available at a limited number of facilities around the world, and, as a result, getting beamtime for SAS experiments at those facilities is so competitive that one can only perform a few experiments per year. In such situation, any attempts to make SAS measurements more efficient are of significance for advances in microstructural studies, especially regarding data-driven materials science.

There are some strategies for efficient use of valuable beamtime at large experimental facilities, from costly hardware upgrades to software-level improvements such as automatic calibration and sample alignment. Among them, estimation of the reasonable measurement time is one which has been overlooked for a long time. Usually, researchers determine measurement time of SAS experiments (or any types of counting experiments, perhaps) empirically to get statistically reliable data, which is often based on a visual impression of data or a rule of thumb taught by their supervisors or instrument scientists. While this practice has supposedly worked so far to assure data quality, it does not mean that there is no need for improvement. As far as we know, there are two papers about measurement time optimisation in terms of the ratio between time lengths of sample and background measurements: Steinhart and Pleštil proposed a special sequence of sample and background measurements to optimise a trade-off between accuracy and measurement time length^12^ and Pauw and Tardif showed the statistical benefit of the optimisation clearly using a simple expression^13^. This paper aims to show that long-ignored spatial correlation of SAS data can give us statistical benefit leading to more efficient use of beamtime at large facilities. We also propose a figure of statistical merit for finishing a measurement at a reasonable time length, which is easy to introduce into real-time monitor systems at beamlines. We focus on SANS in this study because measurement time is much longer for SANS, typically lasting for a few minutes to several tens of minutes, than for synchrotron SAXS which often takes only a few seconds. However, the idea we propose here is so simple that most of the experiments using a two-dimensional detector can benefit from it with appropriate modification and consideration for each use case.

## Signal and Noise in Two-Dimensional Detector

**Figure 1:**
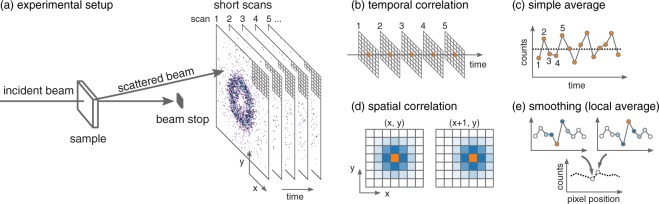
(**a**) Configuration of SANS experiments in this paper and (**b**–**e**) two correlations in a 2D detector and schematics of noise reduction averaging. Broken lines in (**c**) and (**e**) represent averaged values making use of each correlation. Bluish colours indicate a spatial correlation of the orange pixel. The lattice in (**a**) does not represent real pixel size.

In SAS experiments, incident x-ray or neutron beam is scattered by a sample and photons or neutrons are counted by a two-dimensional (2D) detector placed behind the sample (Fig. 1(a)). Scattering intensity distribution obtained with a 2D detector is essentially a histogram with pixels as bins. Counts at each pixel include intrinsic signals from the sample and various noises from many sources such as intrinsic Poisson noise on the signal, extrinsic background intensity from sample environment and neighbouring beamlines, and noises from electrical circuits. Radiation counts follow Poisson statistics where a ratio between standard deviation and observed count *N* is $\frac{1}{\sqrt{N}}$. This ratio means that accumulating counts using longer measurement time practically reduces fluctuation from the “true” count. While the noise reduction effect originates from the nature of Poisson statistics, it is similar to noise-cancelling effect for Gaussian noise which has no temporal correlation. On the other hand, the intrinsic signal from the sample has a strong temporal correlation and should be constant if the sample is at equilibrium. Conventionally, thanks to the absence of temporal correlation in the noise and its presence in the signal, statistical quality of radiation counting measurements is ensured by sufficient measurement time (Fig. 1(b) and (c)).

Behind the obvious and straightforward benefit of the noise reduction effect using temporal correlation, its counterpart has been ignored for long, at least for SAS experiments. Except for sharp peaks such as diffraction, the intensity distribution in SAS experiments is mostly continuous and only shows humps or weak oscillation in log-log plots. In other words, counts at adjacent pixels have spatial correlation for most of SAS experiments. The spatial correlation is expected only for intrinsic signals from a sample but not for noises as we discussed for temporal correlation, and local averaging will cancel out the noises in the same way as a long measurement (Fig. 1(d) and (e)). Combining both noise reduction effects is expected to improve counting statistics more efficiently. The combination is possible and reasonable with physically appropriate smoothing which takes instrumental resolution into account, and it will shorten measurement time for collecting SAS data which is as statistically sufficient as those collected with a long measurement strategy. The details are given in Methods.

## Results

**Figure 2:**
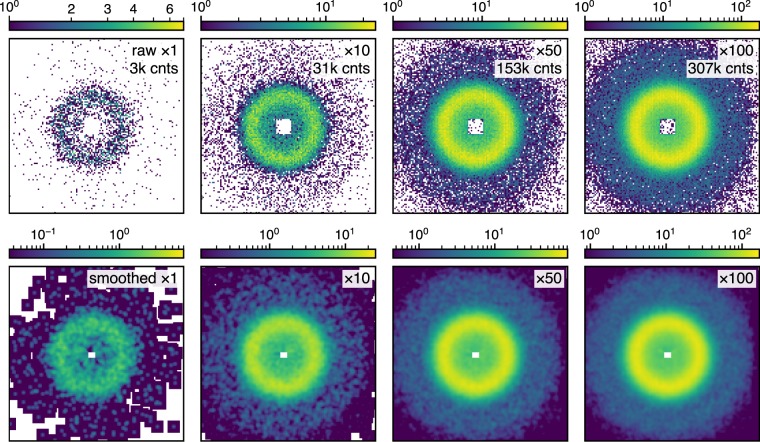
Examples of raw and smoothed SANS patterns for different lengths of measurement time. The sample is silica. Wavelength and the detector distance are 0.6 nm and 18 m, respectively. White pixels represent zero-count (null) pixels. The upper limit for colour bars is equal for each column. The lower limit for raw data is set to 1, while that for smoothed data is set to 0.5% of the upper limit to keep green colour for the middle range intensity from dominating the figures.

We measured a hundred short scans for several samples with different wavelengths and detector distances to see how smoothing works in different types of SANS patterns. Measurement time per short scan was selected so that the total counts of a hundred scans become 3 × 10^5^, a typical and purely empirical rule-of-thumb criterion to terminate a scan. Figure 2 shows examples of raw and smoothed SANS patterns of silica for four different measurement time lengths. We use a logarithmic scale for intensity colouring due to the multiscale nature of SAS intensity. Zero count pixels are displayed with white colour to illustrate their population explicitly, in contrast to a conventional colouring rule to make them indistinguishable from the smallest non-zero count. A remarkable number of null pixels remain even for the longest measurement with ~3 × 10^5^ total counts. These null pixels disappear immediately after smoothing with 9 × 9 Gaussian kernel (see Methods for details).

To see how better data quality becomes after smoothing, we introduce two measures both showing how close a dataset is to another one, i.e., “an answer”. One is the normalized Euclidean distance between two datasets from a detector of *n* × *n* pixels considered as two points in *n*^2^ dimensional space,

1 $$||A-B||=\sqrt{\sum _{i,j}{(\frac{{A}_{ij}-{B}_{ij}}{{\sigma }_{A,ij}})}^{2}},$$

where *A* is the average of a hundred scans as an answer and *B* is the average of a shorter scan dataset with or without smoothing. The standard deviation of the answer dataset, *σ*_*A*,*ij*_, is used as a weight for each pixel. Subscripts *i* and *j* specify pixels in the detector and summation is taken for *i* and *j*. The other measure is Kullback–Leibler (KL) divergence which quantifies the similarity between two probability distributions,

2 $${D}_{{\rm{K}}{\rm{L}}}(P|Q)=\sum _{i,j}{P}_{ij}\,{\rm{l}}{\rm{o}}{\rm{g}}(\frac{{P}_{ij}}{{Q}_{ij}})=\sum _{i,j}{P}_{ij}({\rm{l}}{\rm{o}}{\rm{g}}\,{P}_{ij}-\,{\rm{l}}{\rm{o}}{\rm{g}}\,{Q}_{ij}),\,{\rm{w}}{\rm{h}}{\rm{e}}{\rm{r}}{\rm{e}}\,{P}_{ij}=\frac{{A}_{ij}}{\sum {A}_{ij}}\,{\rm{a}}{\rm{n}}{\rm{d}}\,{Q}_{ij}=\frac{{B}_{ij}}{\sum {B}_{ij}}.$$

Although it is possible to define Euclidean distance with *P*_*ij*_ and *Q*_*ij*_ instead of *A*_*ij*_ and *B*_*ij*_, we limit the use of probability distributions only when they are required for simplicity. For both measures, zero-count pixels in an answer dataset are omitted from the calculation because we cannot define their weights.

**Figure 3:**
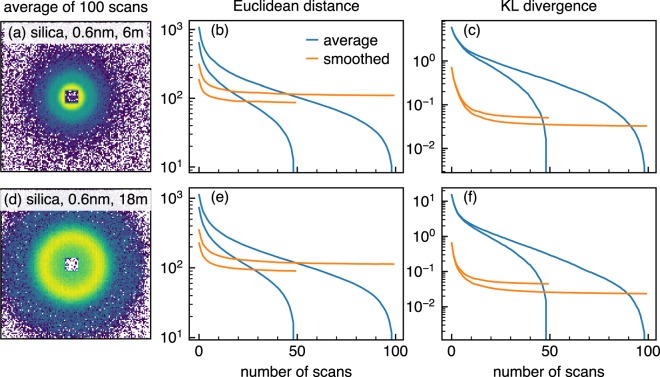
SANS pattern of 100 scans, Euclidean distance, and Kullback-Leibler divergence of the silica sample measured at 6 m ((**a**–**c**)) and 18 m ((**d**–**f**)) detector distances. The number of scans shown in the horizontal axis corresponds to measurement time. Two cases with different answer datasets (50 and 100 scans) are shown as two pairs of blue and orange lines in each figure. Texts in (**a**) and (**b**) indicate the sample name, wavelength, and detector distance, respectively. The same rule applies to the following figures.

Figure 3 shows the measures using two answers (the averages of fifty and a hundred scans) for two different scattering patterns from the same silica sample measured with different conditions. A sudden decrease is seen in both measures for non-smoothed datasets (blue curves) because the datasets approach an answer. Both measures demonstrate the benefit of smoothing defined as a range where orange curves are below blue curves, but with a quantitative difference. Considering that smoothed ten scan data in Fig. 2 looks already similar to the sum of one hundred scans which is one of the answers in Fig. 3 and that smoothed data do not seem less similar to the answer than non-smoothed data after fifty scans (not shown), it is reasonable to conclude that KL divergence captures the benefit of smoothing better than Euclidean distance at least in 2D plots. This is a good example showing that visual impression is still powerful to spot an obvious flaw in data analysis, even though in a study aiming to eliminate the ambiguity of visual impression from measurement. However, KL divergence also has a disadvantage. Two cases with different answers show that the benefit of smoothing indicated by an intersection of orange and blue lines depends on an answer, which makes it difficult to evaluate the benefit quantitatively. The ambiguity is a general disadvantage of measures requiring an answer. On top of that, those measures cannot evaluate data quality during a measurement to determine when to finish it.

**Figure 4:**
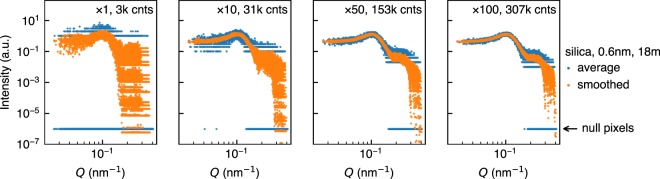
Scatter plots of the pixel-wise mean intensity on a log-log scale. *Q* for the horizontal axis denotes the absolute value of a scattering vector of a pixel which is essentially the distance from the beam centre. Null pixels in non-smoothed data (blue dots) are also shown by adding a small offset value. The beamstop is masked both in smoothing and plotting.

The intrinsic effect of smoothing on 2D pixel data is noise reduction by local averaging. Evaluating noise level from scatter plots of pixel-wise intensity as a function of the distance from the beam centre is a straightforward way to assess the noise reduction effect of smoothing. Figure 4 shows that the effect is apparent. While non-smoothed intensities of multiple short scans are spreading in the vertical directions especially for high *Q*, smoothed intensities sit in a narrow range already for ten scans. The variance for a high *Q* range in non-smoothed data where most counts are one digit remains comparable to their counts even after a hundred scans with 3 × 10^5^ total counts due to the nature of Poisson statistics. Also, it seems that the variance, for both non-smoothed and smoothed data, shows little improvement after fifty scans, indicating that the rule-of-thumb criterion that we chose causes oversampling and that additional measurement time does not yield statistical improvement effectively after the total counts exceed 1.5 × 10^5^. Together with the considerable number of remaining null pixels, these findings suggest that defining the longest measurement as an answer is a naive idea.

**Figure 5:**
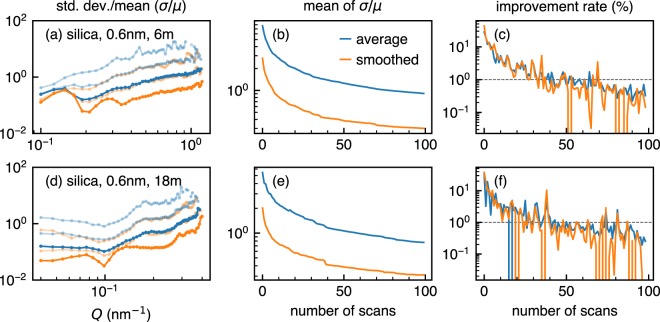
Standard deviation (*σ*) of counts in an annulus normalised by their mean value (*μ*) as a function of scattering vector *Q* which is equivalent to the radius of the annulus (left), the mean of *σ*/*μ* for whole *Q* range as a function of the number of scans (middle), and the improvement rate of the mean of *σ*/*μ* (right). Fifty *Q* ranges equally divided in linear scale are used as rings. Three lengths of measurement time (1, 10, and 100 scan(s) from top to bottom without offset, respectively) are shown for both non-smoothed (blue) and smoothed data (orange) in the left figures. Lines with fainted colours with a lower alpha value indicate one and ten scan(s). Broken lines at 1% in the right figures show a rule-of-thumb criterion to finish a measurement proposed in this study.

To assess the statistical benefit that we can expect from additional measurement time, we show how the standard deviation of counts within “a ring” on the detector improves through a measurement in Fig. 5. The noise reduction effect of smoothing that we see in Fig. 4 is quantitatively shown in Fig. 5(a) and (d). From the figures, it is possible to say that smoothing reduces standard deviation to the level of non-smoothed data with ten times longer measurement time. This is also demonstrated by Fig. 5(b) and (e) showing how the mean of standard deviation for fifty rings improves through a hundred scans. From the two figure, it seems that the pace of improvement does not change whether data is smoothed or not. This becomes more evident when one calculates the relative variation of mean standard deviation between *N* and *N* + 1 scans, which we call an improvement rate, as shown in Fig. 5(c) and (f). In other words, statistical benefit per short scan does not vary with smoothing, which is no surprise because for both cases the statistical improvement relies on the same set of raw counts. The improvement rate confirms our previous visual impression that the statistical quality does not improve significantly after fifty scans, where the rate becomes below 1%. It is true that one can still earn 10% improvement by accumulating minor gain less than 1%. However, statistical benefit per unit measurement time continuously decreases as measurement time gets longer, which means cost-benefit performance also decreases for both users and large experimental facilities. Except for the cases where users have clear statistical criteria for their specific measurements, it would be reasonable to finish a measurement when the improvement rate repeatedly marks below a specific criterion, for example, 1%. Although the criterion is still a rule of thumb, it is much better than wasting precious beamtime by blindly following an unreasonable criterion which causes oversampling.

## Discussion

Image smoothing with a kernel function is mathematically a convolution. Smoothing is also called as blurring or filtering in the field of computer vision where various kernels are employed for different purposes. In research fields where imaging data have a crucial role, such as cell biology and observational astronomy, convolution and deconvolution are frequently employed. Generally speaking, smoothing refers to a data processing to make 1D or 2D data look nice or to remove small fluctuations from data to obtain better results in following workflows such as binarisation in image processing. When a kernel (a window or a filter, depending on fields) is adequately normalised as in this paper, it is equal to what is called kernel density estimation (KDE) in statistics^14^. Because KDE is an essential nonparametric tool in statistics, there are various literatures mathematically ensuring favourable properties of KDE such as consistency, unbiasedness, and efficiency, which are hardly found in the context of smoothing^15–19^. Another advantage of KDE over smoothing is bandwidth selection or optimisation^20–22^, although we don’t put emphasis on it in this work. As expected from its name, KDE estimates the “true” probability density distribution from sampling results. These benefits of KDE are available if we take SAS intensity distribution as sampling results of the “true” probability density distribution determined by a sample and experimental conditions. Although KDE sounds more scientifically legitimate, we use “smoothing” which may be a familiar concept to most of readers.

**Figure 6:**
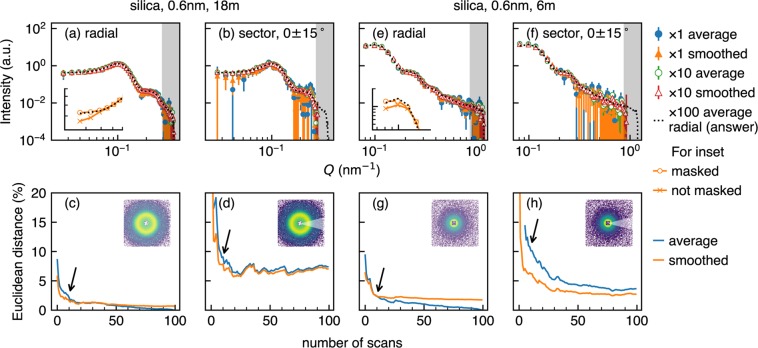
Radial and sector averages of one short scan and ten scans (upper row) and Euclidean distances from the radial average of a hundred scans (lower row) in per cent. Data of the silica sample for 18 m ((**a**)–(**d**)) and 6 m ((**e**)–(**h**)) detector distances are used. For Euclidean distances calculation, we use logarithmic intensity and black shaded parts on the right side of the upper figures were ignored. Error bars are calculated using pyFAI^24^. Beamstops were masked in both smoothing and averaging. White areas in insets in the lower row show averaged ranges. Black arrows indicate the position of 10 scans shown in the upper row. Note that error bars for smoothed data were calculated with a conventional uncertainty propagation using square roots of smoothed intensities as standard deviations. Insets in (**a**) and (**e**) show the effect of masking on the lowest *Q* range in the main figures. Black broken line is “the answer” from a hundred scans without smoothing. Both vertical and horizontal axes in the insets are in a logarithmic scale.

**Figure 7:**
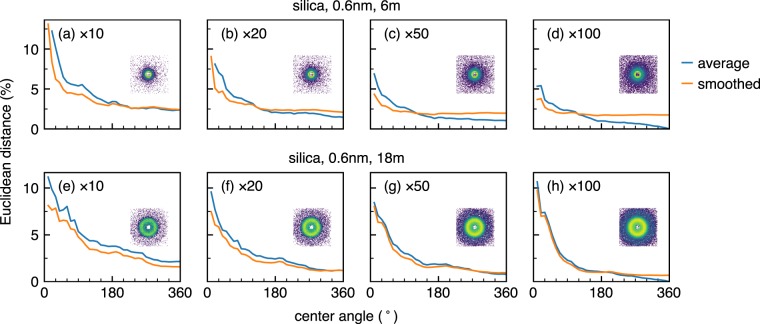
Variation of Euclidean distances as a function of centre angle in sector averaging for different lengths of measurement time. Blue and orange lines denote simple average and smoothed data, respectively as in other figures. The full radial average of the simple average for a hundred scans is taken as an answer. Inset figures show how 2D scattering patterns look like for each measurement time.

Smoothing an intensity distribution obtained with a 2D detector can be interpreted in two ways: the introduction of spatial correlation among neighbouring pixels on the detector or the local averaging which smears counts on neighbouring pixels. Smoothing cancels out the heterogeneous detection efficiency of pixels and all kinds of noises such as Poisson noise and electrical noise by local averaging and reduces the variance in the intensity distribution as shown in Figs 4 and 5. In real analysis, the subject of model fitting is a radially averaged 1D intensity dataset as a function of the scattering vector *Q*. In fact, radial (or circular) averaging, which is indeed a form of smoothing, has a remarkable noise reduction effect which overwhelms our smoothing procedure. For example, if one simply needs radially averaged 1D intensity, only one short scan with poor statistical quality (see Figs 2 and 4) may be enough to reproduce most features in the longest scans as shown in Fig. 6(a) and (e). This surprising result comes from the fact that large counts of inner pixels compensate small sampling numbers for inner annuli and, conversely, large sampling numbers for outer annuli compensate small counts of outer pixels. That is, smoothing does not have an advantage regarding radial averages. Euclidean distances shown in Fig. 6(c) and (g) confirm that improvement by smoothing is negligible for short measurements. On the other hand, there is a noticeable improvement in the Euclidean distance for sector averages in Fig. 6(h). The enhancement can be attributed to an additional local average effect by smoothing which compensates weak noise reduction ability of sector averaging. Figure 7 quantitatively shows the benefit of smoothing in sector average. For example, the sector average of smoothed data with twenty scans (orange lines in Fig. 7(b) and (f)) has comparable data quality to that of the simple average of a hundred scans (blue lines in Fig. 7(d) and (h)) when the centre angle is less than 60°, suggesting that significant reduction in measurement time is achievable for anisotropic samples. Stable benefits of smoothing shown in Fig. 7(e) to (h) indicate that a minor improvement in Fig. 6(d) compared to Fig. 6(h) is caused by a statistical fluctuation. It should be noted that the applicability of smoothing depends on the type of intensity distribution and instrumental resolution. If resolution is high and there is an intrinsic sharp structure, the benefit of smoothing will be limited.

The effect of beamstop masking is worth mentioning. Insets in Fig. 6(a) and (e) show how beamstop masking in smoothing affects the lowest *Q* range in 1D intensity plots. While unmasked smoothing causes underestimated intensities at the lowest *Q* points, no severe data deterioration is observed for masked smoothing. This is realised by intensity interpolation for masked pixels using intensities of unmasked pixels.

Uncertainty is important in model fitting to weight data points. However, we have not reached a solution to incorporate the smearing effect by smoothing into the propagation of uncertainty. The simplest estimate of propagated standard deviation after smoothing ${\hat{\sigma }}_{ij}$, assuming that the pixel intensities follow independent Poisson statistics, can be calculated with

3 $${\hat{\sigma }}_{ij}=\sqrt{\sum _{m,n}{(K(|{r}_{ij}-{r}_{mn}|){\sigma }_{mn})}^{2}},$$

where $K(|r|)$ is a smoothing kernel and *σ*_*mn*_ is a standard deviation of a pixel (*m*, *n*) inside the kernel before smoothing which is equal to the positive square root of summed counts $\sqrt{{v}_{mn}}$ of *N* short scans divided by *N*. For most of pixels, Eq. 3 gives the ratio ${\sigma }_{ij}/{\hat{\sigma }}_{ij}\sim 4$. This seemingly significant improvement may not be true for smeared regions due to underestimated ${\hat{\sigma }}_{ij}$ which does not include the degree of smearing. Smearing effect depends on the shape of intensity distribution and is not straightforward to integrate with uncertainty propagation. This lack of rigorous uncertainty estimation may be unfavourable for those who need careful model fitting with reasonable weights. A recent study has shown that there is an upper bound in difference between KDE and the true probability density distribution, which might be useful for uncertainty estimation^23^.

There is always a trade-off between smoothing ability, or measurement time reduction ability, and the level of data deterioration. We conclude, however, the trade-off brings no severe consequences at least for SANS data if one uses a moderate kernel bandwidth. Experimenters may have a concern about missing “true” features by smoothing which a long measurement might have given. Using a proper kernel bandwidth small enough compared to the direct beam relieves the concern, which is realised by using single pixel bandwidth for most SANS experiments with the direct beam of a few or several pixels on a side. It is also true that a small feature that would be smeared out by such moderate smoothing is intrinsically difficult to confirm even with a long measurement. Another concern about the loss of the lowest *Q* points due to data deterioration by smearing out pixels inside and outside the beamstop has been resolved by masked smoothing. Regarding resolution, smearing by smoothing has a minor effect in SANS data compared to instrumental resolution mainly determined by direct beam size. Therefore, no significant impact of smearing is expected on model fitting for the case of SANS except for uncertainty estimation. This might not apply to SAXS data or any other data with high resolution, and a careful test with a standard sample will be needed.

Measurement time reduction by smoothing described above might only have a small impact in total because there are often other time-consuming processes such as sample changing and wait time to stabilise temperature or magnetic field. Additionally, while the deterioration at the lowest *Q* range is well suppressed by beamstop masking, smearing still affects sharp structures and, probably, anisotropic features as well if a kernel is isotropic. However, it certainly has a positive impact on researchers who want to conduct SANS experiments more efficiently with precise temperature step or a large set of samples. Other possible situations where smoothing has an advantage are 1) salvaging anisotropic data with poor statistics for sector average, 2) time-resolved stroboscopic measurements, and 3) lab SAXS with weak incident beam intensity. Smoothing works as a noise reducer for high *Q* ranges as well where most features are hidden in the forest of error bars usually, which brings a benefit for neutron reflectometry experiments. Similarly, unfavourable effects for low frequency in analyses using Fourier transformation such as real space simulation can be avoided by smoothing as well. As smoothed data is a linear sum of Gaussian functions, smooth derivative functions for any points become available, which may lead to a new analysis strategy for SAS data. Further amelioration is feasible such as anisotropic kernels and adaptive kernel bandwidths following *Q* resolution. As for a criterion to terminate a measurement, using the intensity variance is a naive idea which should be replaced by a physical quantity of interest such as a particle size and other microstructural information.

In the recent new framework of materials science exploiting computational resources and machine learning, the value of experimental datasets is becoming unprecedentedly high. This study is an attempt to take back beamtime lost by naively following empirical criteria. Ironically, this type of efforts may deteriorate user-friendliness guaranteed by the low throughput of neutron experiments. However, in the aforementioned trend, any approaches to accelerate measurement throughput and increase the number of datasets obtained within limited beamtime would be of importance. Our study demonstrates the possibility of data quality improvement at large experimental facilities without huge investments on hardwares. We believe that there is still room for improvement in conventional data processing workflows, which is achievable by introducing statistical techniques used in other fields.

## Methods

### Experimental setups

SANS experiments were conducted at SANS-I installed at a spallation neutron source SINQ, Paul Scherrer Institute, Switzerland. Table 1 summarises measured samples and experimental conditions. Note that only selected results for silica with the density of 36 vol% are shown in figures because two conditions for the silica sample are enough to demonstrate the advantages and disadvantages of smoothing on SANS data. Pixel dimensions are 7.5 × 7.5 mm^2^.

**Table 1:** Samples and experimental conditions. Sample parameters indicate particle diameters for polystyrene and vol% with the same radius ~60 nm for silica. Measurement time for 100 scans include IO overhead about 2 sec/scan. The second column shows the detector distance defining *Q* range together with wavelength. Total counts are sum of intensities of all pixels on the detector.

samples		polystyrene			silica		
sample parameters		50 nm	220 nm	10 *μ*m	0.5	5	36
wavelength (nm)		0.6	1.7	1.7	0.6	0.6	0.6
1 scan (sec)	6 m	25	60	—	10	3.5	0.4
	18 m	15	30	60	8.7	1.6	0.3
100 scan (min)	6 m	50	105	—	20	9	5
	18 m	33	65	100	17	6	4
total counts	6 m	300k	90k	—	160k	300k	300k
	18 m	300k	280k	25k	290k	300k	300k

### Smoothing or kernel density estimation

A set of pixel intensity values, $\{{v}_{ij}\}(i,j=\mathrm{1,}\cdots ,N)$, detected by a 2D square detector of *N* × *N* pixels, is a histogram generated from a probability density function *P*(*r*) for pixel positions ${r}_{ij}(i,j=\mathrm{1,}\cdots ,N)$. *P*(*r*) represents an intrinsic signal from the sample and is determined by various factors such as sample composition, measurement conditions, and instrument setups. If the pixel size is small enough, which is valid for SAS experiments, a constant value *P*_*ij*_ can represent detection probability for a pixel at *r*_*ij*_. Given that *P*(*r*) is only defined within the detector, the sum of *P*_*ij*_ for all pixels is equal to unity, yielding

4 $$\int P(r)dr={\int }_{{\rm{\det }}}P(r)dr=\sum _{i,j}{P}_{i,j}=1.$$

A normalised intensity at a pixel is defined as a sum of *P*_*ij*_ and deviation *ε*_*ij*_,

5 $$\frac{{v}_{ij}}{M}={P}_{ij}+{\varepsilon }_{ij},$$

where $M=\sum {v}_{ij}$ is total counts within the detector for a given measurement time. *ε*_*ij*_ consists of all kinds of deviation from *P*_*ij*_ such as Poisson noise, detection efficiency for the pixel, and electric noises in circuits, and its sum for the detector is expected to be zero. Standard deviation of Poisson noise as a major uncertainty component in *v*_*ij*_ is $\sqrt{{v}_{ij}}$, and its ratio to observed counts $\frac{1}{\sqrt{{v}_{ij}}}$ decreases with accumulating *v*_*ij*_. Therefore, one can consider a pixel intensity dataset {*v*_*ij*_} collected for sufficient measurement time as a good estimation of *P*(*r*). This is a conventional strategy to improve the signal-to-noise ratio by only using the temporal correlation of signals. The strategy is always confronted with a trade-off between measurement time and estimation accuracy, especially when one uses limited beamtime at large experimental facilities. To relax the limitation, we focus on the spatial correlation of signals. One can expect a strong spatial correlation between *P*_*ij*_ and those at adjacent pixels because *P*(*r*) is a smooth function for most of the cases. On the other hand, *ε*_*ij*_ does not have spatial correlations except detection efficiency. By taking local average with appropriate weights, noises without spacial correlation can be suppressed significantly.

Mathematically, a weighted local average of 2D pixel datasets corresponds to discrete convolution

6 $${\hat{P}}_{ij}=\sum _{m,n}{P}_{mn}K(|{r}_{ij}-{r}_{mn}|),$$

where *K*(*r*) is a kernel function defining local weights and $|{r}_{ij}-{r}_{mn}|$ is pixel distance between two pixels located at *r*_*mn*_ and *r*_*ij*_. This procedure is called with various ways such as filtering, blurring, or smoothing in the field of computer vision, or image processing which sounds more familiar. While these names are focused on the effect of the procedure, people in statistics call it kernel density estimation which focuses on a functional side of the procedure. The difference in the terminologies is not surprising if one considers what each field emphasises. In this paper, we use smoothing which is much easier for general readers to understand what we do here. It is worth noting, however, that smoothing is a statistically reasonable method to estimate a probability density function if a proper kernel is used, not a dubious data-manipulation technique just to make data neat.

We introduced a Python library Astropy for smoothing, specifically Gaussian2DKernel for a normalised isotropic Gaussian kernel and convolve for smoothing. The dimensions of the kernel were 9 × 9 pixels and the bandwidth was set to one pixel. We omitted a standard data-reduction workflow such as background subtraction and detection efficiency correction to evaluate the effect of smoothing on simple averaged counts to avoid the complexity of error estimation. Unlike other uncertainty factors, detection efficiency shows spatial correlation with a lattice structure reflecting the physical configuration of the detector components. Therefore, the efficiency correction should be performed before smoothing to obtain the accurate intensity distribution in the standard workflow. In this work, detection efficiency is smoothed out in the same manner as Poisson and circuit noises. Linear scale counts were used for convolution while visualisation by contour plots is based on a logarithmic scale. The beamstop was masked in convolution. We used interpolate option in convolve with which masked pixels were interpolated with the intensities of unmasked pixels within the range of a kernel matrix. Note that the kernel is renormalised if interpolation is needed. Due to the interpolation, pixels near the edge of the beamstop has artificial intensity after smoothing which appears as a shrinking of white beamstop in Fig. 2. These pixels were ignored in all calculations by using beamstop masks. Calculation time was in the order of 10^−2^ s for each convolution of 128 × 128 array with 9 × 9 kernel using a laptop PC, which is short enough for on-site monitoring. One may think that the polar coordinate system is more appropriate than the Cartesian coordinate system considering the symmetry of SAS data. However, transforming uniformly located pixel data in Cartesian coordinates into those in polar coordinates inevitably involves local approximation, which obscures the effect of smoothing. Thus, we intentionally use Cartesian coordinates to assess the smoothing technique properly.

## Supplementary information

Supplementary Information

Figure S1
